# Draft Genome Sequences of Two Lactobacillus johnsonii and Three Ligilactobacillus salivarius Strains Isolated from Intestinal Microbiomes of Chickens

**DOI:** 10.1128/mra.00925-21

**Published:** 2022-02-03

**Authors:** Alyxandra Reed, Magdalena A. Olszewska, Amy Mann, Estefanía Novoa Rama, Harshavardhan Thippareddi, Manpreet Singh, Henk C. den Bakker

**Affiliations:** a Center for Food Safety, Department of Food Science and Technology, University of Georgia, Griffin, Georgia, USA; b Department of Food Science and Technology, University of Georgia, Athens, Georgia, USA; c Department of Poultry Science, University of Georgia, Athens, Georgia, USA; d Department of Industrial and Food Microbiology, Faculty of Food Science, University of Warmia and Mazury in Olsztyn, Olsztyn, Poland; University of Maryland School of Medicine

## Abstract

This report describes the genome sequences of two Lactobacillus johnsonii strains (AER105 and AER25) and three Ligilactobacillus salivarius strains (AER35, AER36, and AER04) recovered from broiler chicken gastrointestinal tracts in the southeastern United States. These genome sequences will enhance our understanding of the ecology of lactobacilli in the chicken gut microbiome.

## ANNOUNCEMENT

As part of a project aiming to characterize lactobacilli found in the chicken microbiome, we sequenced five strains—two Lactobacillus johnsonii (strains AER105 and AER25) and three Ligilactobacillus salivarius (strains AER35, AER36, and AER04). All strains were isolated from the ilea of five individual chickens from farms in the southeastern United States. The ilea and their contents were homogenized in sterile buffered peptone water (BPW; Difco, Sparks, MD) using a stomacher, and 10 μL of the homogenate was used to inoculate MRS broth (AcuMedia, Lansing, MI) and incubated at 37°C for 24 h under either aerobic or elevated CO_2_ (5% CO_2_) conditions. After incubation, approximately 10 μL of the liquid MRS broth was streaked on MRS agar plates and incubated under similar conditions as the broth. After 24 h, an individual colony was picked from the plate and cultured for further experiments. Prior to whole-genome sequencing, isolates were confirmed to be *Lactobacillus* or *Ligilactobacillus* through Gram staining and partial 16S rRNA sequencing. All isolates described here were recovered from aerobic cultures, with the exception of L. johnsonii AER25, which was isolated from a culture incubated under 5% CO_2_ conditions.

DNA was isolated using a DNeasy blood and tissue kit (Qiagen) using 1 mL of a liquid (MRS) culture incubated overnight at 37°C and treated with 180 μL of lysozyme (20 mg/mL; Sigma-Aldrich, St. Louis, MO) for 60 min. Whole-genome sequencing was performed on the MiSeq platform using a Nextera XT kit (Illumina, San Diego, CA) and a V2 kit (Illumina) to obtain 2 × 250-bp paired-end reads. Paired-end reads were used in Shovill v1.1.0 (T. Seemann; https://github.com/tseemann/shovill) to create draft genomes. Within the Shovill pipeline, SPAdes v3.15.3 (1, 2) was used as the genome assembler, and Trimmomatic v0.39 (3) was used to trim the reads before assembly. Contigs with a size of less than 200 bp were excluded from the assembly.

The assembly sizes of the L. johnsonii draft genome sequences were 1,869,983 bp (AER25) and 1,799,247 bp (AER105), while the assembly sizes for the *L. salivarius* draft genome sequences were 1,927,325 bp (AER35), 1,950,306 (AER36), and 2,083,167 (AER04). One intact prophage region was identified by PHASTER (https://phaster.ca/; accessed 26 August 2021 [4]) in all genomes, except for L. johnsonii AER25. Plasmid-derived contigs were identified with MOB-suite software (5, 6) and BLASTN v2.10.0+ (7) in all draft genomes, except for L. johnsonii AER25. No overlap between prophage regions and plasmid-derived contigs was found. Annotation was performed using NCBI’s PGAP annotation pipeline (8); an overview of the number of coding sequences can be found in Table 1. Bacteriocins are small proteins with antimicrobial properties. The number of predicted bacteriocin-encoding genes as inferred by the PGAP annotation pipeline was smaller in the L. johnsonii genomes than in the *L. salivarius* genomes (see Table 1).

**TABLE 1:** Isolates and assembly statistics

Strain	Species	SRA no.	GenBank accession no.^*a*^	No. of read pairs	Total assembly size (bp)	No. of contigs	*N*_50_ (bp)	*N*_90_	No. of CDS^*b*^	GC content (%)	No. of bacteriocins^*b*^
AER25	Lactobacillus johnsonii	SRR15667827	JAINRK010000000	1,361,187	1,869,983	121	46,840	15,668	1,812	34.45	1
AER105	Lactobacillus johnsonii	SRR15667803	JAINRL010000000	756,800	1,799,247	94	103,933	14,372	1,752	34.37	2
AER04	*Ligilactobacillus salivarius*	SRR15667868	JAINRM010000000	1,527,866	2,083,167	164	67,515	14,649	2,032	32.76	5
AER35	*Ligilactobacillus salivarius*	SRR15667869	JAINRN010000000	1,194,030	1,927,325	133	62,999	15,668	1,870	32.98	5
AER36	*Ligilactobacillus salivarius*	SRR15667874	JAINRO010000000	1,101,897	1,950,306	143	59,497	17,726	1,898	32.97	6

a These whole-genome shotgun projects have been deposited at DDBJ/ENA/GenBank under the accession numbers JAINRK000000000, JAINRL000000000, JAINRM000000000, JAINRN000000000, and JAINRO000000000. The versions described in this paper are found in this column.

b CDS, coding DNA sequences. As inferred by NCBI’s PGAP annotation pipeline.

### Data availability.

The genome sequencing and assembly projects have been deposited in DDBJ/EMBL/GenBank under BioProject numbers PRJNA758401, PRJNA758405, PRJNA758411, PRJNA758413, and PRJNA758415. See Table 1 for the SRA and GenBank accession numbers.
